# M2 Macrophage and Extracellular Matrix Genes Are Enriched in High-Activity Lichen Planopilaris

**DOI:** 10.1155/drp/5545886

**Published:** 2025-05-29

**Authors:** Ümmügülsüm Yıldız-Altay, Laura J. Burns, Li-Chi Chen, Himanee Parag Dave, Mariko R. Yasuda, Jillian M. Richmond, Maryanne M. Senna

**Affiliations:** ^1^Department of Dermatology, Yale Medical School, New Haven, Connecticut 06510, USA; ^2^Department of Dermatology, UMass Chan Medical School, Worcester, Massachusetts 01605, USA; ^3^Department of Dermatology, Massachusetts General Hospital, Boston, Massachusetts 02114, USA; ^4^Harvard Medical School, Boston, Massachusetts 02115, USA; ^5^Department of Dermatology, Lahey Hospital and Medical Center, Burlington, Massachusetts 01803, USA

**Keywords:** cicatricial alopecia, fibrosis, hydroxychloroquine, laser, lichen planopilaris, macrophage polarization, NB-UVB

## Abstract

The pathophysiology of lichen planopilaris (LPP), a lymphocytic primary cicatricial alopecia, is largely unknown. We evaluated RNA expression of lesional scalp biopsies taken before and after 6 months of treatment monotherapy with oral hydroxychloroquine (HCQ), narrow band ultraviolet B (NB-UVB), or low level laser light therapy (LLLLT). *PTGER4* and *DOCK2* were significantly increased in all patients after treatment. *CYP1A2*, a drug metabolism enzyme, and *SSR2*, a gene involved in B cell activation and maturation, were increased posttreatment for the HCQ arm. *VEGFA*, which has been reported to be downregulated by phototherapy was decreased post NB-UVB treatment, while *SAA1*, an apolipoprotein gene present in plasma that is upregulated in response to tissue injury, was increased posttreatment for the NB-UVB arm. No significant differentially expressed genes (DEGs) in the LLLLT arm before and after treatment. The expressions of *CD68*, *COL5A1*, *MMP9*, *COL6A3*, and *CD44* were significantly higher at the baseline in biopsies from patients with a Lichen Planopilaris Activity Index (LPPAI) score ≥ 4 compared with those with an LPPAI < 4. These genes are involved in extracellular matrix organization and M2, or profibrotic, macrophage polarization, which is congruent with follicular scarring. Our data identify potential RNA biomarkers of LPPAI and suggest that M2 macrophages may play a role in LPP immunopathogenesis.

## 1. Introduction

Lichen planopilaris (LPP) is a lymphocytic cicatricial alopecia that may clinically manifest as a single lesion or multifocally anywhere on the scalp. Symptoms such as itching, burning, and tenderness often accompany hair loss [1]. The disease is characterized by infundibular inflammation leading to destruction of pilosebaceous units and replacement with fibrosis [2]. Sebaceous glands, the key contributors in hair cycling and biology, are destroyed in LPP [2–5]. Women are more frequently affected than men, with a reported female-to-male ratio of approximately 4.9:1 for classic LPP [6].

While the full etiology of LPP remains unknown, several hypotheses have been proposed including bulge immune privilege collapse, sebaceous gland dysfunction, and peroxisome proliferator-activated receptor-γ (PPARγ) deficiency [7–12]. Current evidence suggests that LPP results from CD8^+^ T cell-driven destruction of epithelial stem cells. This theory alone, however, does not explain the associated fibrosis [8, 12, 14].

Our study objective was to characterize differential expression of fibrosis- and inflammation-related genes in LPP scalp biopsies before and after treatment. The questions we addressed in this study were (a) how do expression levels of specific genes correlate with disease activity levels in LPP at the baseline? and (b) how does gene expression change after three different interventions (hydroxychloroquine [HCQ], narrow band ultraviolet B [NB-UVB], and low level laser light therapy [LLLLT])?

## 2. Methods

### 2.1. Study Population and Treatment Allocation

This study was conducted with Institutional Review Board approval. Fifteen Caucasian patients (Supporting Table 1) (14 female and 1 male, mean age: 64 years [range, 23–75]) with biopsy-proven LPP were recruited to participate and all patients received treatment, given as monotherapy for 6 months by randomization into 3 different groups: oral HCQ (5 mg/kg/ABW day, *n* = 5 patients), NB-UVB phototherapy (thrice weekly, *n* = 6 patients), or LLLLT (thrice weekly, *n* = 4 patients).

All patients recruited self-identified as Caucasian. While this limits generalizability, the study aimed to minimize interindividual variability due to genetic background and focus on treatments effects.

### 2.2. Disease Activity Assessment

In 2009, Chiang et al. developed the Lichen Planopilaris Activity Index (LPPAI) to establish a numeric summary of disease activity (range: 0 [no clinical evidence of disease activity]–10 [maximal disease activity]) [15]. We used this score as well as clinical grading of the extent of perifollicular erythema and scaling observed at the biopsy site pre- and posttreatment to categorize each subject's degree of active disease as high or low.

### 2.3. Tissue Collection and RNA Extraction

Punch biopsies (4 mm) were obtained from lesional scalp tissue before and after treatment. Samples were formalin-fixed and paraffin-embedded (FFPE). Vertical sections were sequentially stained with H&E to confirm visualization of the terminal hair follicle and bulge. FFPE tissues were then processed for RNA extraction using the QIAGEN RNeasy FFPE kit, following the manufacturer's protocol.

### 2.4. Gene Expression Profiling

RNA expression data for the NanoString nCounter Sprint Profiler system, utilizing Human Fibrosis Panel (770 genes) along with 16 additional genes of interest (see Supporting Table 2). Raw data were processed and normalized by using NanoString's nSolver 4.0 digital analyzer software. Fold change calculation and statistical analyses were also performed using the Advanced Analysis Module. Normalization was performed using a set of 10 housekeeping genes: *PPIA*, *PGK1*, *NOL7*, *GUSB*, *ARMH3*, *CNOT10*, *ACAD9*, *RPLP0*, *MTMR14*, *and NUBP1.* Differential gene expression was calculated by nSolver software using three built-in methods: the NanoString error model, standard *t*-tests, and discovery rate (FDR) correction. To account for multiple testing, nSolver applies the Benjamini–Yekutieli procedure, which controls the FDR under dependency structures. The *p* values generated by this method were used to identify statistically significant genes. Cell type scores were also computed by nSolver as the geometric mean of log2-transformed normalized counts across cell type-specific marker gene sets.

All expression data are deposited on GEO database under accession # GSE235392.

### 2.5. Data Analysis

All data were analyzed with GraphPad Prism 9 software (GraphPad Software, Inc). Statistical significance was considered at *p* ≤ 0.05. Each figure legends included individual statistical methods. H&E specimens were additionally evaluated by a blinded dermatopathologist and hair specialist (MRY and MMS), and the degree of inflammation (none, mild, and moderate to severe), fibrosis (present/absent), and destruction of sebaceous glands (present/absent) were scored for each specimen. The results of the blinded H&E grading of scalp specimens correlated with the clinical scoring of patients into high and low activity categories.

## 3. Results

**Figure 1:**
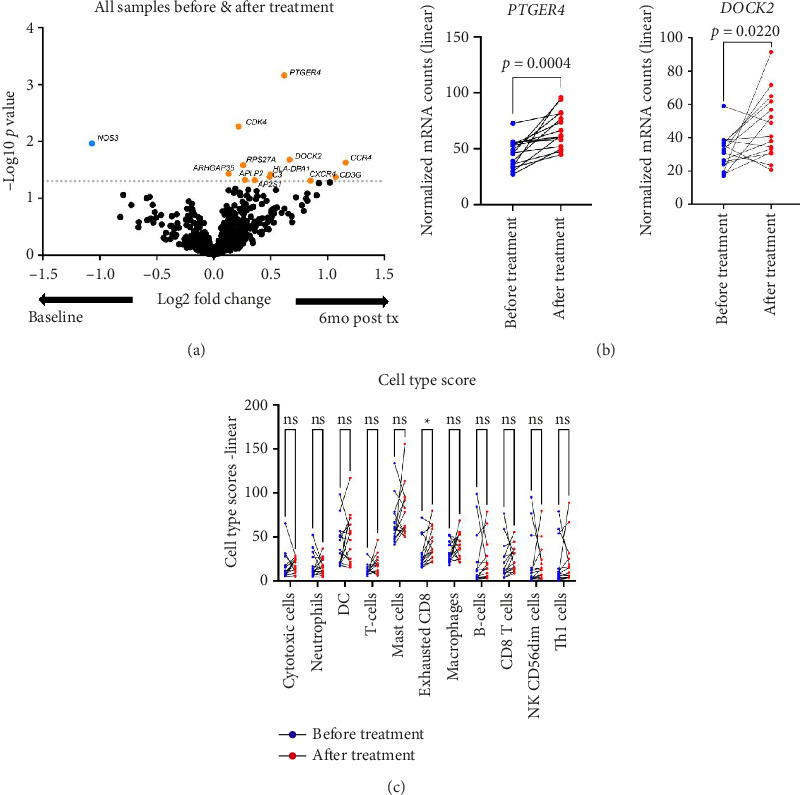
Differential gene expression in LPP scalp biopsies before and after treatment reveals *PTGER4* and *DOCK2* as potential biomarkers of treatment responsiveness, with concomitant increases in exhausted CD8+ T cells. (a) Volcano plot of differentially expressed genes before versus after treatment (dashed line = *p* < 0.05). (b) Paired analysis of normalized RNA counts of top DEGs revealed that *PTGER4* (*p*=0.0004) and *DOCK2* (*p*=0.022) were significantly higher post-treatment. (c) Multiple paired *t* tests with *p* value corrections of the cell type profiling scores of each patient before and after treatment revealed that normalized scores for exhausted CD8 cells were significantly higher after treatment (^∗^*p*=0.018676).

In this study, the efficacy of the three treatment arms, namely, oral HCQ, NB-UVB, or LLLLT, was evaluated in patients, with HCQ showing partial to full responses, while LLLLT and NB-UVB were only able to induce partial responses. Patient demographics and LPPAI scores before and after treatment are summarized in Supporting Table 1. To better understand the impact of treatment on disease, we performed differential gene expression analysis of all biopsies before and after each treatment. We found 13 genes reaching *p* < 0.05 values: upregulated genes were C-C motif chemokine receptor 4 (*CCR4*), CD3 gamma subunit of T-cell receptor complex (*CD3G*), and one downregulated gene, nitric oxide synthase 3 (*NOS3*) (Figure 1(a)). We performed paired *t*-test by subject on a subset of differentially expressed genes (DEGs) identified in the unbiased analysis (Figure 1(b)), including prostaglandin E receptor 4 (*PTGER4*) and dedicator of cytokinesis 2 (*DOCK2*) genes. We also performed cell-type profiling, which revealed higher exhausted CD8 T cells after treatments (Figure 1(c)).

**Figure 2:**
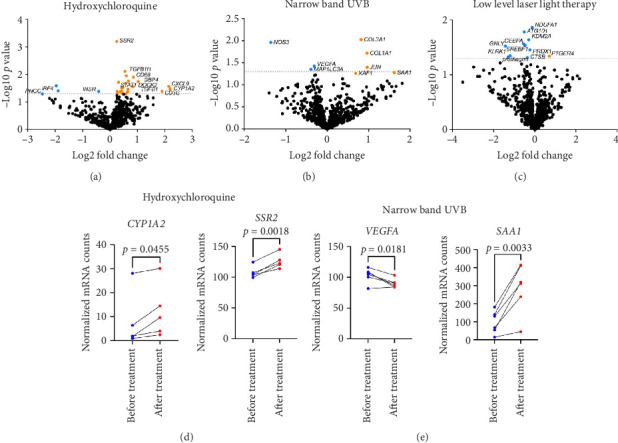
Differential gene expression in each treatment arm at the baseline and 6 months reveals genes related to treatment mechanism of action are changed accordingly. Differentially expressed genes were calculated for before versus after treatment with (a) hydroxychloroquine, (b) narrow band ultraviolet B, or (c) low level laser light therapy (dashed line at *p*=0.05). (d) Normalized gene counts for *CYP1A2* and *SSR2* were significantly higher after treatment with hydroxychloroquine (paired *t*-tests *p*=0.0455 and *p*=0.0018 respectively). (e) Normalized gene counts for *VEGFA* were significantly lower after treatment with narrow band ultraviolet B*. SAA1* was significantly higher after treatment with narrow nand ultraviolet B (paired *t*-tests *p*=0.0181 and *p*=0.0033 respectively). DEGs in the low level laser light device arm had low log fold changes and were ns on paired *t* tests.

Next, we analyzed each treatment arm separately, comparing gene expression before and after treatments within each group (Figure 2(a)). There were zero overlapping significant DEGs between treatment arms. We performed paired t-tests for top DEGs for each arm to confirm directionality. Cytochrome P450 Family 1 Subfamily A Member 2 (*CYP1A2*) and Signal Sequence Receptor Subunit 2 (*SSR2*) genes were elevated posttreatment with HCQ. Serum amyloid A1 (*SAA1*) gene was increased and vascular endothelial growth factor A (*VEGFA*) gene was decreased after NB-UVB therapy (Figure 2(b)). There were no significant genes for LLLLT before versus after treatment after performing paired *t*-test for top DEGs.

**Figure 3:**
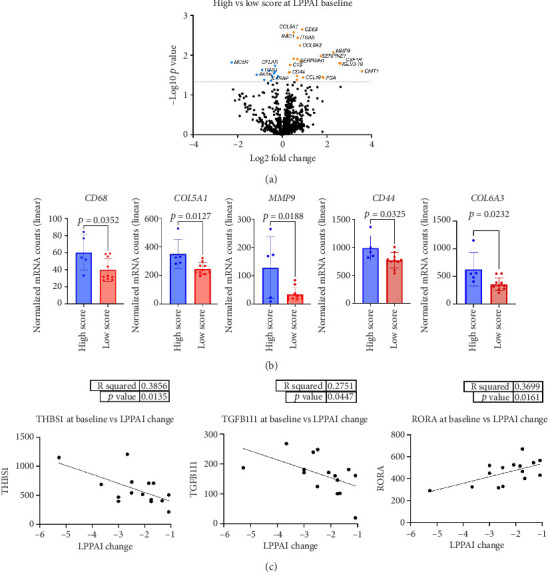
Comparison of differential gene expression between high and low LPPAI scores at the baseline and linear regression analysis for baseline gene expression associated with LPPAI change. (a) Differentially expressed genes in LPPAI high score as compared with low score at baseline using a clinical score of 4 as a cutoff (*p* < 0.05). (b) Normalized gene counts for *CD68*, *COL5A1*, *MMP9*, *CD44*, and *COL6A3* were significantly higher in high versus low score biopsies (unpaired *t*-tests, *p*=0.0352, 0.0127, 0.0188, 0.0232, 0.0163,  *and* 0.0325, respectively). (c) Linear regression analysis revealed the genes *THBS1* and *TGFB1l1* were increased in patients who had a better LPPAI change whereas *RORA* expression was higher in patients who had less LPPAI change.

Next, we investigated whether disease severity using the LPPAI correlated with specific genes at the baseline (Figure 3(a)). The cutoff value of LPPAI was set at 4, with severe disease being defined as LPPAI scores of ≥ 4. There were 7 upregulated genes and 1 downregulated gene that achieved *p* values < 0.05 (Table 1). Genes were analyzed individually and were significantly elevated in high LPPAI score biopsies (Table 2 and Figure 3(b)).

Lastly, we performed linear regression analyses for LPPAI changes before versus after treatment (Figure 3(c)). Thrombospondin-1 (*THBS1*) and Transforming Growth Factor Beta-1-Induced Transcript 1 *(TGFB1l1)* genes were increased with greater reductions of LPPAI score. Patients with lower LPPAI reductions had higher *RORA* expression.

## 4. Discussion

Our investigation into LPP gene expression has revealed a complex transcriptome that likely relates to its phenotypically diverse presentations and outcomes. Among the notable findings in our patient cohort, the most significantly upregulated gene in the pooled posttreatment group was *PTGER4,* which is a subtype of Prostaglandin E receptor [16]. The PGE2-EP4 signaling pathway, which binds to PTGER4 receptor, is known to enhance the expansion and activation of Th17 cells stimulated by IL-23 leading to inflammation in ankylosing spondylitis, atopic dermatitis, psoriasis, and alopecia areata [17, 18]. In addition, EP4 receptor signaling plays a critical role in dendritic cell maturation and migration [16] and initiation of skin immune responses with migration/maturation of Langerhans cells [19]. It is unclear how this pathway may be involved in resolution of inflammation in LPP. DOCK-2 was also significantly upregulated in the pooled posttreatment group. DOCK-2 is expressed on lymphocytes and macrophages and has a role in the differentiation of natural killer T cells, type 2 T helper cells, and plasmacytoid dendritic cells [20]. DOCK-2 is induced by TGF-β and contributes to fibrosis in idiopathic pulmonary fibrosis [21]. In the context of our study, this could indicate a wound healing response after the inflammatory response has stopped. Alternatively, it could indicate that the treatments did not fully prevent fibrosis.

Our study uncovered some intriguing insights into the pharmacogenomic aspects of some LPP therapies. Posttreatment elevation of *CYP1A2*, a drug metabolism enzyme activated by N-demethylation [22], suggests a metabolic response to treatment, particularly with HCQ. Similarly, *SSR2*, expressed in the endoplasmic reticulum (ER) [23] and involved in B cell activation and maturation [24], was also elevated post-HCQ treatment. *VEGFA* was decreased post-NB-UVB treatment, which has been similarly seen to be decreased by light therapy [25] and in psoriasis patients after NB-UVB therapy [26]. *SAA1*, an apolipoprotein gene upregulated in response to tissue injury [27], exhibited increased expression post-NB-UVB treatment. In contrast, the LLLLT group did not exhibit significant changes in gene expression posttreatment.

Among the three interventions, both NB-UVB and HCQ demonstrated reductions in fibrosis-related gene expressions, while LLLLT did not yield significant changes. The decrease in *VEGFA* and increase in *SAA1* following NB-UVB suggests a potential role in modulating fibrotic and repair pathways. These findings imply that NB-UVB or HCQ may be more effective than LLLLT in preventing progression to fibrosis in LPP, though larger studies are needed to confirm this.

While we did not observe changes in the total amount of macrophages present in scalp biopsies based on cell type scores, our data revealed higher expression of M2 macrophage-associated genes at the baseline in patients with high LPPAI scores, indicating alternative polarization in lesional scalp. M2, or “alternatively activated,” macrophages are considered “profibrotic” rather than “proinflammatory.” Their role has been well characterized in scleroderma, a heterogeneous group of autoimmune fibrosing skin disorders [28–31] and, more recently, has been implicated in LPP pathogenesis as M2 macrophages were shown to microscopically differentiate LPP from its clinical variant, frontal fibrosing alopecia [32].

In addition to M2 macrophage signatures, extracellular matrix (ECM) genes were significantly expressed, which may relate to fibrosis and/or disease activity. *COL6A3*, an ECM component and M2 macrophage-related gene identified in our study, has been implicated in conditions like diabetic retinopathy [33]. Similarly, *COL5A1*, involved in collagen fibril organization, was upregulated in central centrifugal cicatricial alopecia (CCCA) [34]. *MMP9*, which is significantly higher in patients with a high LPPAI score, along with *COL5A1* and *CD44*, regulate endodermal cell differentiation and ECM remodeling [34, 35]. Targeting *MMP9* may offer a therapeutic strategy for LPP.

Our data suggest a positive association between higher baseline *THBS1* and *TGFB1l1* expression and LPPAI changes. *THBS1* plays a role in wound healing via TGF-β activation and ECM remodeling [36], while *TGFB1l1* influences androgen responsiveness and increases intrinsic TGF-β within fibrous scar-producing myofibroblasts [37]. These may be important prognostic biomarkers though they would need to be validated in a larger patient cohort.

## 5. Conclusions

In conclusion, our study provides insights into the multifaceted mechanisms underlying LPP pathogenesis and treatment response. From the dysregulation of immune signaling pathways to pharmacogenomic variations and macrophage polarization, our findings shed light on potential therapeutic targets and avenues for further investigation in the management of LPP and related conditions [13].

## Figures and Tables

**Table 1:** Differential gene expression analysis comparing high versus low LPPAI.

Gene symbol	Gene name	Log2 fold change	−Log10 *p* value
MMP9	Matrix metallopeptidase 9	2.29	2.06499685
SERPINE1	Serpin family E member 1	1.73	1.9788107
IGLV3-19	Immunoglobulin lambda variable 3–19	2.57	1.80134291
CSF1R	Colony stimulating factor 1 receptor	2.62	1.79048499
CHIT1	Chitinase 1	3.58	1.59516628
FASN	Fatty acid synthase	−1.14	1.51004152
HAVCR1	Hepatitis A virus cellular receptor 1	1.82	1.44977165
FGA	Fibrinogen alpha chain	1.84	1.43415218

**Table 2:** Differential gene expression analysis of genes was analyzed individually in high LPPAI score biopsies.

Gene symbol	Gene name	Log2 fold change	−Log10 *p* value
CHIT1	Chitinase 1	3.58	1.59516628
CD68	CD68 molecule	0.899	2.64589156
COL5A1	Collagen type V alpha 1 chain	0.513	2.57839607
COL6A3	Collagen type VI alpha 3 chain	0.796	2.24641694
CD44	CD44 molecule	0.336	1.57511836

## Data Availability

The data have been deposited in the Gene Expression Omnibus (GEO) under the accession number GSE235392 and can be accessed at https://www.ncbi.nlm.nih.gov/geo/query/acc.cgi.

## Supporting Information

Supporting Information 1 Supporting Table 1: Patient demographics, treatment allocation, and LPPAI scores before and after treatment.

Supporting Information 2 Supporting Table 2: Probe annotation details for the NanoString Human Fibrosis Panel 770 genes and 16 additional custom-selected genes.
