# Supplementary material for: M2 Macrophage and Extracellular Matrix Genes Are Enriched in High-Activity Lichen Planopilaris
Source: Dermatol Res Pract. 2025 May 29;2025:5545886. doi: 10.1155/drp/5545886 (PMC12140823; doi:10.1155/drp/5545886)
Supplement: Supporting Information 1 — Supporting Table 1: Patient demographics, treatment allocation, and LPPAI scores before and after treatment. [file 5545886.f1.docx]

| **Subject ID** | **Gender** | **Age** | **Race** | **Ethnicity** | **Treatment** | **LPPAI Score 0** | **LPPAI Score 6** | **LPPAI change** | **Responder** |
| --- | --- | --- | --- | --- | --- | --- | --- | --- | --- |
| BG02 | F | 53 | White | Non-Hispanic | NB-UVB | 3.83 | 1.33 | -2.5 | Partial |
| BG03 | F | 66 | White | Non-Hispanic | NB-UVB | 7.6 | 2.33 | -5.27 | Partial |
| BG04 | F | 71 | White | Non-Hispanic | NB-UVB | 3.49 | 0.83 | -2.66 | Partial |
| BG06 | F | 62 | White | Non-Hispanic | LLLLT | 5.16 | 2.66 | -1.33 | Partial |
| BG07 | F | 82 | White | Non-Hispanic | LLLLT | 4.16 | 1.67 | -2.49 | Partial |
| BG09 | F | 67 | White | Non-Hispanic | NB-UVB | 6.16 | 2.5 | -3.66 | Partial |
| BG10 | F | 73 | White | Non-Hispanic | LLLLT | 4.08 | 2 | -2.08 | Partial |
| BG12 | M | 59 | White | Non-Hispanic | NB-UVB | 3.08 | 1.33 | -1.75 | Partial |
| BG15 | F | 70 | White | Non-Hispanic | NB-UVB | 2.08 | 1 | -1.08 | Partial |
| BG16 | F | 75 | White | Non-Hispanic | HCQ | 2.5 | 0.66 | -1.84 | Partial |
| BG17 | F | 67 | White | Non-Hispanic | HCQ | 3.5 | 0.5 | -3 | Full |
| BG22 | F | 23 | White | Non-Hispanic | HCQ | 1.08 | 0 | -1.08 | Full |
| BG23 | F | 55 | White | Non-Hispanic | HCQ | 2.42 | 0.67 | -1.75 | Partial |
| BG39 | F | 64 | White | Non-Hispanic | LLLLT | 3.67 | 0.67 | -3 | Partial |
| BG40 | F | 68 | White | Non-Hispanic | HCQ | 1.83 | 0.17 | -1.66 | Full |

**Supplemental Table 1**

**Patients Demographics, Treatment modalities, and LPPAI Scores Before and After Therapy**

This table summarize demographics information, treatment groups, and clinical response of all 15 patients enrolled in the study. LPPAI scores are shown at baseline (Month 0) and after 6 months of treatment (Month 6), along with calculated changes and categorized treatment response.

Abbreviations:

NV-UVB: Narrow Band Ultraviolet B phototherapy

LLLLT: Low-Level Laser Light Therapy

HCQ: Oral Hydroxychloroquine

LPPAI: Lichen Planopilaris Activity Index,

LPPAI Score 0: Baseline score before treatment

LPPAI Score 6: Score after 6 months of treatment
